# Supplementary material for: Modeling neuron-astrocyte interactions in neural networks using distributed simulation
Source: PLoS Comput Biol. 2025 Sep 19;21(9):e1013503. doi: 10.1371/journal.pcbi.1013503 (PMC12494295; doi:10.1371/journal.pcbi.1013503)
Supplement: S1 Appendix — (PDF) [file pcbi.1013503.s001.pdf]

S1 Appendix: Description of the neuron-astrocyte network models

Han-Jia Jiang, Jugoslava Aćimović, Tiina Manninen, Iiro Ahokainen, Jonas Stapmanns, Mikko Lehtimäki, Markus Diesmann, Sacha J. van Albada, Hans Ekkehard Plesser, and Marja-Leena Linne

This appendix contains details of the neuron-astrocyte network models considered in this article, following the convention of previous studies (Nordlie et al., 2009; Senk et al., 2022). Table A contains a general and systematic description of the models in a standardized tabular format summarizing model components, mathematical equations, and inputs and outputs. Tables B and C list specific parameter values of the models. Table B lists parameter values used in the benchmarks of this study. Table C lists parameter values used to construct the *in silico* model based on the experimental study by Pirttimaki et al. (2017). This model reproduces and extends the previously presented analysis of astrocytic role in neuronal synchronization (Pirttimaki et al., 2017). The parameters are carefully fitted to the experimental findings from the literature as described in Methods and Results of the article and S3 Appendix. Notations in Table B and C follow the style of mathematical descriptions in this article, or the style of NEST parameters if there is no corresponding notation in the mathematical descriptions (see S2 Appendix).

Table A: Neuron-astrocyte network model presented in the standardized tabular format.

**Model summary**			
**Populations**	Excitatory neurons (E), Inhibitory neurons (I), Astrocytes (A)		
**Topology**	Random connectivity, no spatial organization		
**Connectivity**			
Neuron-to-neuron	Each pair of neurons is connected with probability p_{primary}.		
Astrocyte-to-neuron, Neuron-to-astrocyte	Each generated excitatory synapse interacts with one selected astrocyte with probability $p_{\mathrm{third\_if\_primary}}$. In case interaction is established, connections from the presynaptic neuron to astrocyte, and from the astrocyte to the postsynaptic neuron are generated.		
**Neuron model**	Adaptive Exponential Integrate-and-fire (AdEx) (Brette and Gerstner, 2005; Naud et al., 2008)		
**Astrocyte model**	$[\mathrm{Ca}^{2+}]$ dynamics according to De Young-Keizer (De Young and Keizer, 1992) and Li-Rinzel (Li and Rinzel, 1994) models; Slow Inward Current (SIC) according to Nadkarni-Jung (NJ) model (Nadkarni and Jung, 2003)		
**Synapse model**			
Neuron-to-neuron, neuron-to-astrocyte	Short-term facilitation and depression according to Tsodyks-Markram model (Tsodyks et al., 1998, 2000). The kinetic is exponentially decaying for neuron-to-neuron interactions, and delta function for neuron-to-astrocyte interactions.		
Astrocyte-to-neuron	Delivers continuous SIC to the target (postsynaptic) neuron		
**External inputs**			
Neuron	Poisson noise that induces postsynaptic current		
Astrocyte	1) Poisson noise that affects IP_3 concentration<br>2) Gaussian noise that affects calcium concentration		
**Populations**			
Name	Elements	Size (no. neurons)	Indices in connectivity matrices $\mathbf{\Gamma}_{\mathrm{NN}}$, $\mathbf{\Gamma}_{\mathrm{NA}}$,$\mathbf{\Gamma}_{\mathrm{AN}}$
Excitatory neurons	AdEx	N_{E}	$i = 1...N_{\mathrm{E}}$
Inhibitory neurons	AdEx	$N_{\mathrm{I}} = N - N_{\mathrm{E}}$	$i = (N_{\mathrm{E}} + 1)...N$
Astrocytes	NJ	N_{A}	$k = 1...N_{\mathrm{A}}$

**Connectivity**		
Source	Target	Pattern
{E,I}	{E,I}	Connectivity matrix $\mathbf{\Gamma}_{\mathrm{NN}} = \{\gamma^{\mathrm{NN}}_{ji}\}_{i,j=1\ldots N}$. $\gamma^{\mathrm{NN}}_{ji} = 1$ if there is a synapse from neuron j to neuron i, $\gamma^{\mathrm{NN}}_{ji} = 0$ otherwise. Each pair of neurons (excitatory and inhibitory) is connected with probability p_{primary}. This results in binomial distribution of inputs and outputs: $\mathbf{B}(N^2, p_{\mathrm{primary}})$.
E	A	Each astrocyte is associated with a set of synapses and receives inputs from the presynaptic neurons of these synapses according to $\mathbf{\Gamma}_{\mathrm{NA}} = \left\{\gamma^{\mathrm{NA}}_{ji,k}\right\}_{k=1\ldots N_{\mathrm{A}}, i=1\ldots N, j=1\ldots N_{\mathrm{E}}}$. $\gamma^{\mathrm{NA}}_{ji,k} = 1$ if an astrocyte receives inputs from a synapse ji, $\gamma^{\mathrm{NA}}_{ji,k} = 0$ otherwise. Each generated synapse can be associated with only one selected astrocyte, and the probability of this association is $p_{\mathrm{third\_if\_primary}}$.
A	{E,I}	Astrocyte outputs to all postsynaptic neurons of its associated synapses according to $\mathbf{\Gamma}_{\mathrm{AN}} = \{\gamma^{\mathrm{AN}}_{k,ji}\}_{k=1\ldots N_{\mathrm{A}}, i=1\ldots N, j=1\ldots N_{\mathrm{E}}}$. $\gamma^{\mathrm{AN}}_{k,ji} = 1$ if the postsynaptic neuron i receives input from the astrocyte k, $\gamma^{\mathrm{AN}}_{k,ji} = 0$ otherwise. For each postsynaptic neuron, the astrocyte pool from which to select the associated astrocyte(s) is confined by two parameters S_{pool} and *pool_type*, which define the pool size and the way to determine the pool, respectively.

Connectivity rules	
1) Primary connectivity rule	The primary connectivity rule is the rule to establish primary (neuron-to-neuron) connections with. Possible rules include `pairwise_bernoulli`, `fixed_indegree`, `fixed_outdegree`, and `fixed_total_number`. For specifications of these rules, see Senk et al. (2022) and NEST documentation for connectivity concepts.
2) Third-factor connectivity rule	The third-factor connectivity rule is the rule to establish third-in (neuron-to-astrocyte) and third-out (astrocyte-to-neuron) connections with. `third_factor_bernoulli_with_pool` is the only rule available. For specifications of this rule, see NEST documentation for `TripartiteConnect()` and `third_factor_bernoulli_with_pool`.

**Neuron model**	
	For each neuron $i = 1 \ldots N$ in the network
1) Subthreshold dynamics	$C_{\mathrm{m}} \cdot \frac{dV_i(t)}{dt} = f(V_i(t)) - W_i(t) + I_{\mathrm{syn},i}(t) + I_{\mathrm{SIC},i}(t) + I_{\mathrm{noise},i}(t)$<br>$f(V_i(t)) = g_{\mathrm{L}} \cdot (E_{\mathrm{L}} - V_i(t)) + g_{\mathrm{L}} \cdot \Delta_{\mathrm{T}} \cdot \exp\left(\frac{V_i(t) - V_{\mathrm{th}}}{\Delta_{\mathrm{T}}}\right)$<br>with membrane capacitance C_{m}, membrane potential V_i, adaptation variable W_i, summed synaptic current $I_{\mathrm{syn},i}$ from all connected neurons and external inputs (see **Synaptic input for neuron**), summed SIC input $I_{\mathrm{SIC},i}$ from all connected astrocytes, external noise current $I_{\mathrm{noise},i}$ (see **External inputs**), leak conductance g_{L}, leak reversal potential E_{L}, slope factor Δ_{T}, and spike initiation threshold V_{th}.
2) Adaptation	$\tau_W \cdot \frac{dW_i(t)}{dt} = a \cdot (V_i(t) - E_{\mathrm{L}}) - W_i(t)$<br>with subthreshold adaptation a and adaptation time constant τ_W.
3) Spiking and reset	Spiking condition: $V_i(t^-_{i,sp}) \leq V_{\mathrm{peak}}$ and $V_i(t_{i,sp}) > V_{\mathrm{peak}}$ with spiking threshold V_{peak} and spike time $t_{i,sp}$ of the spth spike of neuron i.<br>If the spiking condition is met then:<br>1. Save the new spike time: $t_{i,sp} \to \mathcal{S}_i$ ($\mathcal{S}_i$: spikes recorded from neuron i)<br>2. Reset: $V_i(t \in (t_{i,sp}, t_{i,sp} + t_{\mathrm{ref}}]) \leftarrow V_{\mathrm{reset}}, W_i(t^+_{i,sp}) \leftarrow W_i(t^-_{i,sp}) + b$<br>with spike-triggered adaptation b, refractory period t_{ref}, and voltage reset V_{reset}.

**Astrocyte model**	
	For each astrocyte $k = 1...N_{\mathrm{A}}$ in the network
1) $\mathrm{IP_3}$	$\frac{d[\mathrm{IP_3}]_k(t)}{dt} = \frac{[\mathrm{IP_3}]_0 - [\mathrm{IP_3}]_k(t)}{\tau_{\mathrm{IP_3}}} + \Delta_{\mathrm{IP_3}} \cdot J_{\mathrm{syn},k}(t)$ with baseline value $[\mathrm{IP_3}]_0$ of $\mathrm{IP_3}$ concentration, time constant $\tau_{\mathrm{IP_3}}$ of the decay of $\mathrm{IP_3}$ concentration, and parameter $\Delta_{\mathrm{IP_3}}$ determining the increase in $\mathrm{IP_3}$ concentration induced by synaptic input $J_{\mathrm{syn},k}$ (see **Synaptic input for astrocyte**).
2) Calcium	$\frac{d[\mathrm{Ca^{2+}}]_k(t)}{dt} = J_{\mathrm{channel},k}(t) - J_{\mathrm{pump},k}(t) + J_{\mathrm{leak},k}(t) + J_{\mathrm{noise},k}(t)$ with $\mathrm{IP_3R}$-mediated release $J_{\mathrm{channel},k}$ of calcium from ER to cytosol, ATP-dependent pumping $J_{\mathrm{pump},k}$ of calcium from cytosol to ER, leak $J_{\mathrm{leak},k}$ of calcium between ER and cytosol, and random fluctuations $J_{\mathrm{noise},k}$ in calcium dynamics (see **External inputs**).
3) Gating variable for $\mathrm{IP_3R}$ inactivation	$\frac{dh_{\mathrm{IP_3R},k}(t)}{dt} = \alpha_{h,k}(t) \cdot (1 - h_{\mathrm{IP_3R},k}(t)) - \beta_{h,k}(t) \cdot h_{\mathrm{IP_3R},k}(t)$ $\alpha_{h,k}(t) = k_{\mathrm{IP_3R}} \cdot K_{\mathrm{d,inh}} \cdot \frac{[\mathrm{IP_3}]_k(t) + K_{\mathrm{d,IP_{3,1}}}}{[\mathrm{IP_3}]_k(t) + K_{\mathrm{d,IP_{3,2}}}}$ $\beta_{h,k}(t) = k_{\mathrm{IP_3R}} \cdot [\mathrm{Ca^{2+}}]_k(t)$ with fraction $h_{\mathrm{IP_3R}}$ of $\mathrm{IP_3Rs}$ that are not yet inactivated by calcium, two rates $\alpha_{h,k}$ and $\beta_{h,k}$ of the exchange between activated and inactivated $\mathrm{IP_3Rs}$, astrocytic $\mathrm{IP_3R}$ binding constant $k_{\mathrm{IP_3R}}$ for calcium inhibition, astrocytic $\mathrm{IP_3R}$ dissociation constant $K_{\mathrm{d,inh}}$ of calcium (inhibition), and the first and second astrocytic $\mathrm{IP_3R}$ dissociation constants $K_{\mathrm{d,IP_{3,1}}}$ and $K_{\mathrm{d,IP_{3,2}}}$ of $\mathrm{IP_3}$.
4) Channel, pump and leak currents	$J_{\mathrm{channel},k}(t) = r_{\mathrm{ER,cyt}} \cdot v_{\mathrm{IP_3R}} \cdot m^3_{\infty,k}(t) \cdot n^3_{\infty,k}(t) \cdot h^3_{\mathrm{IP_3R},k}(t) \cdot \left([\mathrm{Ca^{2+}}]_{\mathrm{ER},k}(t) - [\mathrm{Ca^{2+}}]_k(t)\right)$ $J_{\mathrm{pump},k}(t) = \frac{v_{\mathrm{SERCA}} \cdot [\mathrm{Ca^{2+}}]^2_k(t)}{K^2_{\mathrm{m,SERCA}} + [\mathrm{Ca^{2+}}]^2_k(t)}$ $J_{\mathrm{leak},k}(t) = r_{\mathrm{ER,cyt}} \cdot v_{\mathrm{L}} \cdot \left([\mathrm{Ca^{2+}}]_{\mathrm{ER},k}(t) - [\mathrm{Ca^{2+}}]_k(t)\right)$ $m_{\infty,k}(t) = \frac{[\mathrm{IP_3}]_k(t)}{[\mathrm{IP_3}]_k(t) + K_{\mathrm{d,IP_{3,1}}}}$ $n_{\infty,k}(t) = \frac{[\mathrm{Ca^{2+}}]_k(t)}{[\mathrm{Ca^{2+}}]_k(t) + K_{\mathrm{d,act}}}$ with ratio $r_{\mathrm{ER,cyt}}$ between astrocytic ER and cytosol volumes, maximum rate $v_{\mathrm{IP_3R}}$ of calcium release via astrocytic $\mathrm{IP_3Rs}$, astrocytic $\mathrm{IP_3R}$ dissociation constant $K_{\mathrm{d,act}}$ of calcium (activation), maximum rate v_{SERCA} of calcium uptake by astrocytic SERCA pumps, half-activation constant $K_{\mathrm{m,SERCA}}$ of astrocytic SERCA pumps, rate constant v_{L} of calcium leak from astrocytic ER to cytosol, and steady-state values m_∞ and n_∞ for two gating variables of $\mathrm{IP_3Rs}$.
5) Calcium concentration in ER	$[\mathrm{Ca^{2+}}]_{\mathrm{ER},k}(t) = \frac{[\mathrm{Ca^{2+}}]_{\mathrm{tot}} - [\mathrm{Ca^{2+}}]_k(t)}{r_{\mathrm{ER,cyt}}}$ with parameter $[\mathrm{Ca^{2+}}]_{\mathrm{tot}}$ determining the maximal cytosolic calcium concentration.

**Synaptic input for neuron**	
Synaptic current	For each neuron $i = 1...N$ in the network, the summed synaptic current is<br><br>$I_{\text{syn},i}(t) = \sum_j \gamma_{ji}^{\text{NN}} \cdot I_{\text{syn},ji}(t) + I_{\text{Poiss},i}(t)$
1) from neurons in the network	$I_{\text{syn},ji}(t) = -g_{\text{exc},ji}(t) \cdot (V_i(t) - E_{\text{exc}}) - g_{\text{inh},ji}(t) \cdot (V_i(t) - E_{\text{inh}})$<br><br>with excitatory and inhibitory synaptic conductances $g_{\text{exc},ji}$ and $g_{\text{inh},ji}$, excitatory and inhibitory reversal potentials E_{exc} and E_{inh}.<br><br>The synaptic conductance is alpha-shaped and modeled with short-term plasticity:<br>$g_{X,ji}(t) = \sum_{sp} w_{X,ji} \cdot u_{ji}(t_{j,sp}) \cdot x_{ji}(t_{j,sp}) \cdot (\frac{t-t_{j,sp}-d_{X,ji}}{\tau_{X,\text{syn}}}) \cdot \exp(1 - \frac{t-t_{j,sp}-d_{X,ji}}{\tau_{X,\text{syn}}})$<br>$\frac{du_{ji}(t)}{dt} = -\frac{u_{ji}(t)}{\tau_{\text{fac}}} + \sum_{sp} U \cdot (1 - u_{ji}(t_{j,sp})) \cdot \delta(t - t_{j,sp} - d_{X,ji})$<br>$\frac{dx_{ji}(t)}{dt} = \frac{z_{ji}(t)}{\tau_{\text{rec}}} - \sum_{sp} u_{ji}(t_{j,sp}) \cdot x_{ji}(t_{j,sp}) \cdot \delta(t - t_{j,sp} - d_{X,ji})$<br>$\frac{dy_{ji}(t)}{dt} = -\frac{y_{ji}(t)}{\tau_{\text{psc}}} + \sum_{sp} u_{ji}(t_{j,sp}) \cdot x_{ji}(t_{j,sp}) \cdot \delta(t - t_{j,sp} - d_{X,ji})$<br>$\frac{dz_{ji}(t)}{dt} = \frac{y_{ji}(t)}{\tau_{\text{psc}}} - \frac{z_{ji}(t)}{\tau_{\text{rec}}}$<br><br>with time constant $\tau_{X,\text{syn}}$ of postsynaptic conductance in the neuron, time constant τ_{psc} of postsynaptic current in the short-term plasticity model, weight $w_{X,ji}$ of neuron-to-neuron connection, release probability u_{ji}, readily releasable fraction x_{ji} of neurotransmitter, active fraction y_{ji} of neurotransmitter, inactive fraction z_{ji} of neurotransmitter, facilitation time constant τ_{fac}, depression time constant τ_{rec}, parameter U determining the increase in u_{ji} with each spike, spike time $t_{j,sp}$ of the spth spike of neuron j, delay $d_{X,ji}$ of neuron-to-neuron connection. $X = \text{exc}$ if $j \leq N_{\text{E}}$ and $X = \text{inh}$ if $j > N_{\text{E}}$.<br><br>Synaptic weights $w_{X,ji}$ and delays $d_{X,ji}$ are supplied to the TBP rule via the `primary` synapse specification.
2) from external input	$I_{\text{Poiss},i}(t)$ (see **External inputs**)
**Synaptic input for astrocyte**	
Synaptic input	For each astrocyte $k = 1...N_{\text{A}}$ in the network, the summed synaptic input is<br><br>$J_{\text{syn},k}(t) = \sum_{ji} \gamma_{ji,k}^{\text{NA}} \cdot J_{\text{syn},ji,k}(t) + J_{\text{Poiss},k}(t)$
1) from neurons in the network	$J_{\text{syn},ji,k}(t) = w_{\text{pre\_to\_astro},ji,k} \cdot \sum_{sp} u_{ji}(t_{j,sp}) \cdot x_{ji}(t_{j,sp}) \cdot \delta(t - t_{j,sp} - d_{\text{pre\_to\_astro},ji,k})$<br><br>where $t_{j,sp}$ is the time of the spth spike of neuron j. The weights $w_{\text{pre\_to\_astro},ji,k}$ and delays $d_{\text{pre\_to\_astro},ji,k}$ of the neuron-to-astrocyte connections are supplied to the TBP rule via the `third_in` synapse specification.
2) from external input	$J_{\text{Poiss},k}(t)$ (see **External inputs**)

Astrocyte-to-neuron current	
Slow inward current (SIC)	For each neuron $i = 1...N$ in the network, the summed SIC input from all connected astrocytes is<br><br>$I_{\mathrm{SIC},i}(t) = \sum_k \sum_j \gamma^{\mathrm{AN}}_{k,ji} \cdot w_{\mathrm{astro\_to\_post},k,ji} \cdot F_{\mathrm{SIC}}\left([\mathrm{Ca}^{2+}]_k(t - d_{\mathrm{astro\_to\_post},k,ji})\right)$<br><br>$F_{\mathrm{SIC}}\left([\mathrm{Ca}^{2+}]_k(t)\right) = a_{\mathrm{SIC}} \cdot \Theta\left(\log(c_{\mathrm{scaled},k}(t))\right) \cdot \log\left(c_{\mathrm{scaled},k}(t)\right)$<br><br>$c_{\mathrm{scaled},k}(t) = \left([\mathrm{Ca}^{2+}]_k(t) - \theta_{\mathrm{SIC}}\right)/\mathrm{nM}$<br><br>where a_{SIC} is the scale of SIC output, θ_{SIC} the calcium threshold for SIC generation, and $\Theta(x)$ the Heaviside step function. The weights $w_{\mathrm{astro\_to\_post},k,ji}$ and delays $d_{\mathrm{astro\_to\_post},k,ji}$ of the astrocyte-to-neuron connections are supplied to the TBP rule via the `third_out` synapse specification.
**External inputs**	
Poisson noise input for neurons	For each neuron $i = 1...N$ in the network, a Poisson noise input is applied:<br><br>$I_{\mathrm{Poiss},i}(t) = -g_{\mathrm{Poiss},i}(t) \cdot (V_i(t) - E_{\mathrm{exc}})$<br><br>$g_{\mathrm{Poiss},i}(t) = \sum_{sp} w_{\mathrm{Poiss},i} \cdot (\frac{t - t_{\mathrm{Poiss},sp} - d_{\mathrm{Poiss}}}{\tau_{\mathrm{exc,syn}}}) \cdot \exp(1 - \frac{t - t_{\mathrm{Poiss},sp} - d_{\mathrm{Poiss}}}{\tau_{\mathrm{exc,syn}}})$<br><br>with Poisson input-induced conductance $g_{\mathrm{Poiss},i}$, weight $w_{\mathrm{Poiss},i}$ of input, spike time $t_{\mathrm{Poiss},sp}$ of the spth spike of input, and delay d_{Poiss} of input. The average firing rate ($\lambda_{\mathrm{Poiss,N},i}$) of the input for each neuron is fixed.
Poisson noise input for astrocytes	For each astrocyte $k = 1...N_{\mathrm{A}}$ in the network, a Poisson noise input is applied:<br><br>$J_{\mathrm{Poiss},k}(t) = w_{\mathrm{Poiss}} \cdot \sum_{sp} \delta(t - t_{\mathrm{Poiss},sp} - d_{\mathrm{Poiss}})$,<br><br>with weight w_{Poiss} of input, spike time $t_{\mathrm{Poiss},sp}$ of the spth spike of input, and delay d_{Poiss} of input. The average firing rate ($\lambda_{\mathrm{Poiss,A},k}$) of the input for each astrocyte is fixed. The Poisson noise input affects the IP_3 dynamics in the astrocyte (see **Astrocyte model** and **Synaptic input for astrocyte**).
Gaussian noise input for neurons	For each neuron $i = 1...N$ in the network, a Gaussian noise current $I_{\mathrm{noise},i}$ is applied. $I_{\mathrm{noise},i}$ has a fixed mean of zero and variance $\sigma_{\mathrm{Gauss,N}}$.
Gaussian noise input for astrocytes	For each astrocyte $k = 1...N_{\mathrm{A}}$ in the network, a Gaussian noise input $J_{\mathrm{noise},k}(t)$ is applied. $J_{\mathrm{noise},k}(t)$ has a fixed mean of zero and variance $\sigma_{\mathrm{Gauss,A}}$. The Gaussian noise input affects the calcium dynamics in the astrocyte (see **Astrocyte model**).

Table B: Parameters of the neuron-astrocyte network models for the benchmarks in this study. All weights and delays of synaptic interactions are listed. For the other parameters, only those that differ from NEST defaults are listed. For a few parameters, values for different models are listed separately. Otherwise, the value applies to all models. The parameters required for the models but not listed here follow defaults of `astrocyte_lr_1994`, `aeif_cond_alpha_astro`, and `tsodyks_synapse` in NEST.

Notation	Value	Description
**Populations**		
N_{E}	$8,000 \times scale$	Number of excitatory neurons
N_{I}	$2,000 \times scale$	Number of inhibitory neurons
N_{A}	$10,000 \times scale$	Number of astrocytes or surrogates
scale	1 (strong-scaling benchmarks)<br>1, 2, 3, 4 (weak-scaling benchmarks)<br>1.25, 2.5, 5, 10, 20, 30, 40, 50 (weak-scaling benchmarks for very large models)	Scale of model
**Connectivity**		
`rule` under `conn_spec`	`pairwise_bernoulli` (benchmarks with neuron-astrocyte network models)<br>`pairwise_bernoulli`, `fixed_indegree`, `fixed_outdegree`, `fixed_total_number` (benchmarks with different primary connectivity rules)<br>`pairwise_bernoulli` (weak-scaling benchmarks with different astrocyte pool sizes)<br>`fixed_indegree` (weak-scaling benchmarks for very large models)	The primary connectivity rule
`rule` under `third_factor_conn_spec`	`third_factor_bernoulli_with_pool`	The third-factor connectivity rule
p_{primary}	0.1 / *scale*	Connection probability between neurons
$p_{\mathrm{third\_if\_primary}}$	0.5	Probability of each created neuron-to-neuron connection to be paired with one astrocyte
S_{pool}	10 (benchmarks with neuron-astrocyte network models, benchmarks with different primary connectivity rules)<br>10, 100, 1000, 10000 (weak-scaling benchmarks with different astrocyte pool sizes)<br>10, 1000 (weak-scaling benchmarks for very large models)	The size of astrocyte pool for each target (postsynaptic) neuron
pool_type	`"random"`	The way to determine the astrocyte pool for each target neuron
**Astrocyte parameters**		
$[\mathrm{IP}_3][0]$	0.4 µM	Initial IP_3 concentration
**`astrocyte_surrogate` parameters (for the "surrogate" model only)**		
SIC output	3.5 pA	Predefined SIC output
**Synaptic interaction parameters**		
w_{exc}	1 nS	Weight of excitatory neuron-to-neuron connections
w_{inh}	4 nS	Weight of inhibitory neuron-to-neuron connections
w_{Poiss}	1 nS	Weight of excitatory noise-to-neuron connections
$w_{\mathrm{pre\_to\_astro}}$	1	Weight of excitatory neuron-to-astrocyte connections (the unit is included in the astrocyte parameter Δ_{IP_3}; see Table A and S2 Appendix)
$w_{\mathrm{astro\_to\_post}}$	0.05 pA	Weight of astrocyte-to-neuron connections

$d_{\mathrm{exc}}, d_{\mathrm{pre\_to\_astro}}$	2 ms	Delay of excitatory neuron-to-neuron and neuron-to-astrocyte connections
$d_{\mathrm{astro\_to\_post}}$	1 ms	Delay of astrocyte-to-neuron connections
d_{inh}	1 ms (all except the "Synchronous" model)<br>2 ms (the "Synchronous" model)	Delay of inhibitory neuron-to-neuron connections
d_{Poiss}	1 ms	Delay of excitatory noise-to-neuron connections
$\tau_{\mathrm{exc,syn}}$	2 ms	Time constant for excitatory connections
$\tau_{\mathrm{inh,syn}}$	4 ms (all except the "Synchronous" model)<br>2 ms (the "Synchronous" model)	Time constant for inhibitory connections
**External input**		
$\lambda_{\mathrm{Poiss,N}}$	2000 spikes/s	Rate of the Poisson noise for each neuron

Table C: Parameters for the models developed to study the impact of neuron-astrocyte interactions on neuronal synchronization. The table lists parameters that were fitted or selected when creating the model, that might differ from the NEST default values. The other parameters required for the models but not listed here follow defaults of `astrocyte_lr_1994`, `aeif_cond_alpha_astro`, and `tsodyks_synapse` in NEST. The same parameters are also listed in the JSON file `neuron_astrocyte_model_parameters.json` in the model implementation code.

Notation	Value	Description
**Populations**		
N_{E}	400	Number of excitatory neurons
N_{I}	100	Number of inhibitory neurons
N_{A}	100	Number of astrocytes
**Connectivity**		
p_{primary}	0.2	Connection probability between neurons
$p_{\mathrm{third\_if\_primary}}$	 0.2<br>0.03	Probability of each created neuron-to-neuron connection to be paired with one astrocyte<br>`pool_type` = `"block"`<br>`pool_type` = `"random"`
S_{pool}	 1<br>5	The size of astrocyte pool for each target neuron<br>`pool_type` = `"block"` (1 astrocyte input per neuron)<br>`pool_type` = `"random"` (max. 5 astrocyte inputs per neuron)
pool_type	`"block"`, `"random"`	The way to determine the astrocyte pool for each target neuron
**Neuron parameters**		
V_{th}	E: -50 mV, I: -52 mV	Spiking initiation threshold
C_{m}	E: 130 pF, I: 104 pF	Cell membrane capacitance
g_{L}	E: 18 nS, I: 4.3 nS	Leak conductance
E_{L}	E: -58 mV, I: -65 mV	Resting potential
Δ_{T}	E, I: 2 mV	Threshold slope factor
τ_W	E: 450 ms, I: 264 ms	Time constant of adaptation current
a	E: 4 nS, I: -0.8 nS	Adaptation parameter
V_{reset}	Gaussian distribution:<br>E, mean: $\bar{V}_{\mathrm{reset}} = -50$ mV, var: $0.05\bar{V}_{\mathrm{reset}}$<br>allowed range of values: $[0.9\bar{V}_{\mathrm{reset}}, 1.1\bar{V}_{\mathrm{reset}}]$<br><br>I, mean: $\bar{V}_{\mathrm{reset}} = -60$ mV, var: $0.05\bar{V}_{\mathrm{reset}}$<br>allowed range of values: $[0.9\bar{V}_{\mathrm{reset}}, 1.1\bar{V}_{\mathrm{reset}}]$	Voltage reset
b	Gaussian distribution:<br>E, mean: $\bar{b} = 300$ pA, var: $0.05\bar{b}$<br>allowed range of values: $[0.9\bar{b}, 1.1\bar{b}]$<br><br>I, mean: $\bar{b} = 130$ pA, var: $0.05\bar{b}$<br>allowed range of values: $[0.9\bar{b}, 1.1\bar{b}]$	Spike triggered adaptation

**Astrocyte parameters**		
$[Ca^{2+}]_{tot}$	Gaussian distribution, mean value fitted to the experimental data: mean: $[\bar{Ca}^{2+}]_{tot} = 1.8958264782990153$ µM, var: $0.1[\bar{Ca}^{2+}]_{tot}$ allowed range of values: $[0.9[\bar{Ca}^{2+}]_{tot}, 1.05[\bar{Ca}^{2+}]_{tot}]$	Total free calcium concentration in terms of cytosolic volume
$[IP_3]_0$	Gaussian distribution, mean value fitted to the data: mean: $[\bar{IP}_3]_0 = 0.010925583871830699$ µM, var: $0.1[\bar{IP}_3]_0$ allowed range of values: $[0.9[\bar{IP}_3]_0, 1.05[\bar{IP}_3]_0]$	Baseline value of IP_3 concentration
$[IP_3][0]$	0.010925583871830699 µM	IP_3 concentration is set to be the same as $[IP_3]_0$ at the start of each simulation
τ_{IP_3}	Gaussian distribution, mean fitted to the data: mean: $\bar{\tau}_{IP_3} = 1058.7589568400845$ ms, var: $0.1\bar{\tau}_{IP_3}$ allowed range of values: $[0.9\bar{\tau}_{IP_3}, 1.05\bar{\tau}_{IP_3}]$	Time constant of the exponential decay of astrocytic IP_3
Δ_{IP_3}	Gaussian distribution, mean fitted to the data: mean: $\bar{\Delta}_{IP_3} = 0.04911592657233997$ µM, var: $0.1\bar{\Delta}_{IP_3}$ allowed range of values: $[0.9\bar{\Delta}_{IP_3}, 1.05\bar{\Delta}_{IP_3}]$	Parameter determining the increase in astrocytic IP_3 concentration induced by synaptic input
a_{SIC}	1	Scaling coefficient, scales all SICs coming from the same astrocyte
**Synaptic interaction parameters (weights)**		
w_{exc}	5 nS	Weight of excitatory neuron-to-neuron connections
w_{inh}	5 nS	Weight of inhibitory neuron-to-neuron connections
w_{Poiss}	1 nS	Weight of excitatory noise-to-neuron and noise-to-astrocyte connections
d_{exc}	1 ms	Delay of excitatory neuron-to-neuron connections
d_{inh}	1 ms	Delay of inhibitory neuron-to-neuron connections
$d_{pre\_to\_astro}$	1 ms	Delay of excitatory neuron-to-astrocyte connections
$d_{astro\_to\_post}$	1 ms	Delay of astrocyte-to-neuron connections
$\tau_{exc,syn}$	2 ms	Time constant for excitatory connections
$\tau_{inh,syn}$	2 ms	Time constant for inhibitory connections
**Synaptic interaction parameters for neuron-astrocyte interaction**		
$w_{pre\_to\_astro}$	Kept equal for all synapses	Weight of excitatory neuron-to-astrocyte interaction
	0.2	`pool_type = "block"`, asynchronous
	0.31	`pool_type = "block"`, network bursting
	1	`pool_type = "random"`, asynchronous
	1.5	`pool_type = "random"`, network bursting (in S3 Appendix)
$w_{astro\_to\_post}$	Kept equal for all synapses	Weight of astrocyte-to-neuron connections
	1 pA	`pool_type = "block"`, asynchronous
	1 pA	`pool_type = "block"`, network bursting
	7 pA	`pool_type = "random"`, asynchronous
	7 pA	`pool_type = "random"`, network bursting (in S3 Appendix)

External inputs, noise		
$\sigma_{\text{Gauss,N}}$	100 pA	Variance of Gaussian noise for neurons
$\sigma_{\text{Gauss,A}}$	0.0003 $\frac{\mu M}{ms}$	Variance of Gaussian noise for astrocytes. Note that parameter is given in $\frac{\mu M}{ms}$ even though the NEST implementation of Gaussian noise usually has the physical unit of pA when used in other models.
$\lambda_{\text{Poiss,E}}, \lambda_{\text{Poiss,I}}$	E: 2700 spikes/s, I: 2500 spikes/s	Rate of the Poisson noise for each neuron
$\lambda_{\text{Poiss,A}}$	4.3281650042083655 Hz<br><br>3.0297155029458556 Hz	Rate of Poisson noise for each astrocyte (fitted to experimental data)<br><br>Rate of Poisson noise, used in all simulations except the one shown in Fig 9 in the main article (reproducing TTX blocking)
**Simulation parameters**		
`pre_sim_time`	20 s	Preliminary simulation that allows model settle in the steady state regime
`sim_time`	5 min	Simulated model time
**Simulation analysis parameters**		
`max_allowed _distance_astro`	2000 ms	Used when detecting calcium transients
`max_allowed _distance_exc`, `max_after_sic_exc`	2000 ms	Used when detecting single-neuron bursts in excitatory neurons
`max_allowed _distance_inh`, `max_after_sic_inh`	400 ms	Used when detecting single-neuron bursts in inhibitory neurons
`hist_window`	2000 ms	Length of a sliding window for binning spike trains when computing correlation coefficients
`hist_shift`	400 ms	Shift of the sliding window

References

R. Brette and W. Gerstner. Adaptive exponential integrate-and-fire model as an effective description of neuronal activity. *Journal of Neurophysiology*, 94:3637–3642, 2005. doi: 10.1152/jn.00686.2005.

G. W. De Young and J. Keizer. A single-pool inositol 1,4,5-trisphosphate-receptor-based model for agonist-stimulated oscillations in Ca^{2+} concentration. *Proceedings of the National Academy of Sciences of the United States of America*, 89(20):9895–9899, 1992. doi: 10.1073/pnas.89.20.9895.

Y.-X. Li and J. Rinzel. Equations for $InsP_3$ receptor-mediated $[Ca^{2+}]_i$ oscillations derived from a detailed kinetic model: a Hodgkin-Huxley like formalism. *Journal of Theoretical Biology*, 166(4):461–473, 1994. doi: 10.1006/jtbi.1994.1041.

S. Nadkarni and P. Jung. Spontaneous oscillations of dressed neurons: a new mechanism for epilepsy? *Physical Review Letters*, 91(26):268101, 2003. doi: 10.1103/PhysRevLett.91.268101.

R. Naud, M. Marcille, C. Clopath, and W. Gerstner. Firing patterns in the adaptive exponential integrate-and-fire model. *Biological Cybernetics*, 99:335–347, 2008. doi: 10.1007/s00422-008-0264-7.

E. Nordlie, M.-O. Gewaltig, and H. E. Plesser. Towards reproducible descriptions of neuronal network models. *PLoS Computational Biology*, 5(8):e1000456, 2009. doi: 10.1371/journal.pcbi.1000456.

T. M. Pirttimaki, R. E. Sims, G. Saunders, S. A. Antonio, N. K. Codadu, and H. R. Parri. Astrocyte-mediated neuronal synchronization properties revealed by false gliotransmitter release. *Journal of Neuroscience*, 37(41):9859–9870, 2017. doi: 10.1523/JNEUROSCI.2761-16.2017.

J. Senk, B. Kriener, M. Djurfeldt, N. Voges, H.-J. Jiang, L. Schüttler, G. Gramelsberger, M. Diesmann, H. E. Plesser, and S. J. van Albada. Connectivity concepts in neuronal network modeling. *PLoS Computational Biology*, 18(9): e1010086, 2022. doi: 10.1371/journal.pcbi.1010086.

M. Tsodyks, K. Pawelzik, and H. Markram. Neural networks with dynamic synapses. *Neural Computation*, 10(4): 821–835, 1998. doi: 10.1162/089976698300017502.

M. Tsodyks, A. Uziel, and H. Markram. Synchrony Generation in Recurrent Networks with Frequency-Dependent Synapses. *Journal of Neuroscience*, 20(1):RC50, 2000. doi: 10.1523/JNEUROSCI.20-01-j0003.2000.
