# Supplementary material for: Modeling neuron-astrocyte interactions in neural networks using distributed simulation
Source: PLoS Comput Biol. 2025 Sep 19;21(9):e1013503. doi: 10.1371/journal.pcbi.1013503 (PMC12494295; doi:10.1371/journal.pcbi.1013503)
Supplement: S2 Appendix — (PDF) [file pcbi.1013503.s002.pdf]

S2 Appendix: Variables and parameters added to the NEST support for astrocytes

Han-Jia Jiang, Jugoslava Aćimović, Tiina Manninen, Iiro Ahokainen, Jonas Stapmanns, Mikko Lehtimäki, Markus Diesmann, Sacha J. van Albada, Hans Ekkehard Plesser, and Marja-Leena Linne

Here, we provide additional details of new developed NEST support for astrocytes to supplement the description given in Methods of the article. The new development implements a model for astrocytic calcium dynamics derived by Li and Rinzel (1994) as a reduction of De Young and Keizer's model (De Young and Keizer, 1992) for calcium in non-excitable cells. The model for astrocyte-neuron interaction through slow inward current (SIC) was published by Nadkarni and Jung (2003). These models have been frequently used in later publications of neuron-astrocyte network models (see Manninen et al., 2018, 2023). The main software components supporting the respective model are illustrated in Fig A. These components are mainly explained in the article and S1 Appendix, and only partly in this appendix.

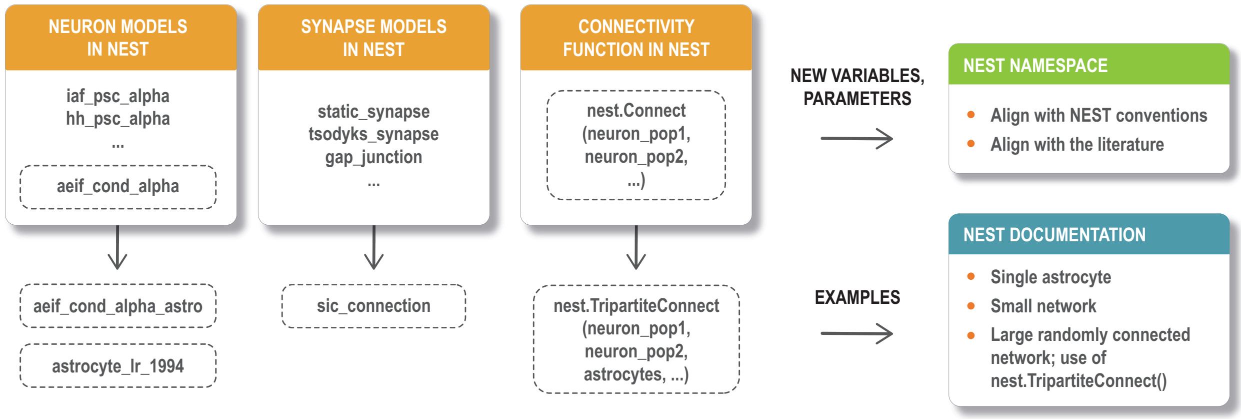

Fig. A: Schematic representation of the software components required for the representation of the models and their embedding in the NEST code. The orange blocks illustrate the added astrocyte-specific models derived from the previously existing NEST models; the old models are listed in boxes, the added models are below the boxes. The green box to the right shows efforts in aligning naming conventions. The blue box below shows examples added to the NEST documentation.

The rest of this appendix describes alignment of naming conventions illustrated in Fig A (green box). Tables A-C compare the parameter and variable names in NEST, the mathematical expressions used in this article, and the mathematical expressions used in publications (Li and Rinzel, 1994; Nadkarni and Jung, 2003) and frequently in later network modeling studies (Manninen et al., 2018, 2023). In addition, the NEST default values (for parameters), the NEST default initial conditions (for variables), the physical units, and description of each variable and parameter are given. **Table A** lists parameters for the new connectivity function `TripartiteConnect()`. **Tables B** and **C** list variables and parameters for the astrocyte model `astrocyte_lr_1994`, respectively. These variables and parameters are harmonized with NEST naming conventions, and thus they differ from the notation used by Li and Rinzel (1994) and Nadkarni and Jung (2003).

References

G. W. De Young and J. Keizer. A single-pool inositol 1,4,5-trisphosphate-receptor-based model for agonist-stimulated oscillations in Ca^{2+} concentration. *Proceedings of the National Academy of Sciences of the United States of America*, 89(20):9895–9899, 1992. doi: 10.1073/pnas.89.20.9895.

Y.-X. Li and J. Rinzel. Equations for $InsP_3$ receptor-mediated $[Ca^{2+}]_i$ oscillations derived from a detailed kinetic model: a Hodgkin-Huxley like formalism. *Journal of Theoretical Biology*, 166(4):461–473, 1994. doi: 10.1006/jtbi.1994.1041.

T. Manninen, R. Havela, and M.-L. Linne. Computational models for calcium-mediated astrocyte functions. *Frontiers in Computational Neuroscience*, 12:14, 2018. doi: 10.3389/fncom.2018.00014.

T. Manninen, J. Aćimović, and M.-L. Linne. Analysis of network models with neuron-astrocyte interactions. *Neuroinformatics*, 21:375–406, 2023. doi: 10.1007/s12021-023-09622-w.

S. Nadkarni and P. Jung. Spontaneous oscillations of dressed neurons: a new mechanism for epilepsy? *Physical Review Letters*, 91(26):268101, 2003. doi: 10.1103/PhysRevLett.91.268101.

Table A: Parameters of the connectivity function `TripartiteConnect()`.

**Parameter in NEST**	**For mathematical descriptions in this article**	**Default value in NEST**	**Description**
`conn_spec`	-	-	Parameters that specify the primary connectivity, i.e., connections between nodes in the two primary populations. See `conn_spec` in the NEST documentation for `Connect()`.
`third_factor_conn_spec`	-	-	Parameters that specify the third-factor connectivity, i.e., connections between primary nodes and nodes in the third population.
`syn_specs`	-	-	Parameters that specify the synaptic interactions in the primary and the third-factor connectivity.
`third_factor_conn_spec` parameters			
`rule`	-	-	The third-factor connectivity rule. At present, `third_factor_bernoulli_with_pool` is the only rule available.
`p`	$p_{\text{third\_if\_primary}}$	-	Probability of each created primary connection to be paired with one third-factor node.
`pool_size`	S_{pool}	Total number of third-factor nodes (if `pool_type` = `"random"`)<br>- (if `pool_type` = `"block"`)	The size of third-factor node pool for each target (postsynaptic) primary node. Each target primary node only receives inputs from the third-factor nodes in this pool.
`pool_type`	*pool_type*	`"random"`	The way to determine the third-factor node pool for each target primary node. If `"random"`, a number (`pool_size`) of third-factor nodes are randomly chosen from all third-factor nodes (without replacement) and assigned as the pool. If `"block"`, the third-factor nodes are evenly distributed to the primary nodes in blocks without overlapping, and the specified `pool_size` has to be compatible with this arrangement.
`syn_specs` parameters			
			Specifications of the synaptic interaction (model, weight, delay, etc.):
`primary`	-	-	– between primary nodes
`third_in`	-	-	– from primary nodes to third-factor nodes
`third_out`	-	-	– from third-factor nodes to primary nodes
			Each follows the style of `syn_specs` in NEST. See `syn_specs` in the NEST documentation for `Connect()`.

Table B: Variables of the astrocyte model `astrocyte_lr_1994`.

Variable in NEST	This article	Original publication	Default initial condition in NEST	Physical unit	Description
`IP3`	$[\mathrm{IP}_3]$	$[\mathrm{IP}_3]$	0.16	µM	IP_3 concentration in the astrocytic cytosol
`Ca_astro`	$[\mathrm{Ca}^{2+}]$	$[\mathrm{Ca}^{2+}]$	0.073	µM	Calcium concentration in the astrocytic cytosol
`h_IP3R`	$h_{\mathrm{IP_3R}}$	q	0.793	-	Fraction of IP_3 receptors on the astrocytic ER that are not yet inactivated by calcium

Table C: Parameters of the astrocyte model `astrocyte_lr_1994`.

Parameter in NEST	This article	Original publication	Default value in NEST	Physical unit	Description
`Ca_tot`	$[Ca^{2+}]_{tot}$	c_0	2	µM	Total free calcium concentration in terms of cytosolic volume
`IP3_0`	$[IP_3]_0$	$[IP_3]^*$	0.16	µM	Baseline value of astrocytic IP_3 concentration
`Kd_act`	$K_{d,act}$	d_5	0.08234	µM	Astrocytic IP_3R dissociation constant of calcium (activation)
`Kd_inh`	$K_{d,inh}$	d_2	1.049	µM	Astrocytic IP_3R dissociation constant of calcium (inhibition)
`Kd_IP3_1`	$K_{d,IP_3,1}$	d_1	0.13	µM	First astrocytic IP_3R dissociation constant of IP_3
`Kd_IP3_2`	$K_{d,IP_3,2}$	d_3	0.9434	µM	Second astrocytic IP_3R dissociation constant of IP_3
`Km_SERCA`	$K_{m,SERCA}$	k_3	0.1	µM	Half-activation constant of astrocytic SERCA pump
`ratio_ER_cyt`	$r_{ER,cyt}$	c_1	0.185	-	Ratio between astrocytic ER and cytosol volumes
`delta_IP3`	Δ_{IP_3}	r_{IP_3}	0.0002	µM	Parameter determining the increase in astrocytic IP_3 concentration induced by synaptic input
`k_IP3R`	k_{IP_3R}	a_2	0.0002	$\frac{1}{\mu M \cdot ms}$	Astrocytic IP_3R binding constant for calcium inhibition
`rate_L`	v_L	v_2	0.00011	$\frac{1}{ms}$	Rate constant of calcium leak from astrocytic ER to cytosol
`rate_IP3R`	v_{IP_3R}	v_1	0.006	$\frac{1}{ms}$	Maximum rate of calcium release via astrocytic IP_3R
`rate_SERCA`	v_{SERCA}	v_3	0.0009	$\frac{\mu M}{ms}$	Maximum rate of calcium uptake by astrocytic SERCA pump
`tau_IP3`	τ_{IP_3}	τ_{IP_3}	7142	ms	Time constant of the exponential decay of astrocytic IP_3
`SIC_th`	θ_{SIC}	0.19669	0.19669	µM	Threshold that determines the minimal level of astrocytic cytosolic calcium sufficient to induce SIC
`SIC_scale`	a_{SIC}	2.11	1	-	Parameter determining the scale of astrocytic SIC output
